# Differential regulation of tissue thiol-disulfide redox status in a murine model of peritonitis

**DOI:** 10.1186/1476-9255-9-36

**Published:** 2012-10-04

**Authors:** Shana M Benton, Zhe Liang, Li Hao, Youngliang Liang, Gautam Hebbar, Dean P Jones, Craig M Coopersmith, Thomas R Ziegler

**Affiliations:** 1 Department of Medicine, Emory University School of Medicine, Atlanta, GA, 30322-0001, USA; 2 Department of Surgery, Emory University School of Medicine, Atlanta, GA, 30322-0001, USA; 3 Center for Clinical and Molecular Nutrition, Emory University School of Medicine, Atlanta, GA, 30322-0001, USA

**Keywords:** Cysteine, Glutathione, Peritonitis, Redox, Sepsis

## Abstract

**Background:**

Glutathione (GSH)/glutathione disulfide (GSSG) and cysteine (Cys)/cystine (CySS) are major redox pools with important roles in cytoprotection. We determined the impact of septic peritonitis on thiol-disulfide redox status in mice.

**Methods:**

FVB/N mice (6–12 week old; 8/group) underwent laparotomy with cecal ligation and puncture (CLP) or laparotomy alone (control). Sections of ileum, colon, lung and liver were obtained and GSH, GSSG, Cys and CySS concentrations determined by HPLC 24 h after laparotomy. Redox potential [E_h_ in millivolts (mV)] of the GSH/GSSG and Cys/CySS pools was calculated using the Nernst equation. Data were analyzed by ANOVA (mean ± SE).

**Results:**

GSH/GSSG E_h_ in ileum, colon, and liver was significantly oxidized in septic mice versus control mice (ileum: septic −202±4 versus control −228±2 mV; colon: -195±8 versus −214±1 mV; and liver: -194±3 vs. -210±1 mV, all P<0.01). Lung GSH/GSSG redox was similar in each group (−191±3 versus −190±2 mV). In contrast, ileal and colonic Cys/CySS E_h_ was unchanged with CLP, while liver and lung Cys/CySS E_h_ became significantly more reducing (liver: septic = −103±3 versus control −90±2 mV; lung: -101±5 versus −81±1 mV, each P<0.05).

**Conclusions:**

Septic peritonitis induced by CLP oxidizes ileal and colonic GSH/GSSG redox but Cys/CySS E_h_ remains unchanged in these intestinal tissues. In liver, CLP oxidizes the GSH/GSSG redox pool and CyS/CySS E_h_ becomes more reducing; in lung, CLP does not alter GSH/GSSG E_h_, and Cys/CySS E_h_ is less oxidized. CLP-induced infection/inflammation differentially regulates major thiol-disulfide redox pools in this murine model.

## Background

Sepsis continues to be a primary cause of morbidity and mortality in critically ill patients in intensive care units worldwide [1,2]. Sepsis, ischemia-reperfusion and inflammatory shock cause oxidative injury to the gut mucosa and depletion of glutathione (GSH) [3,4]. Glutathione (GSH)/glutathione disulfide (GSSG) and cysteine (Cys)/cystine (CySS) are major redox pools with important roles in cytoprotection, cell function, proliferation, apoptosis, and detoxification [5-7]. GSH, and its oxidized form GSSG, are the major low-molecular weight thiol antioxidant couple in cells, and play a major role in antioxidant defense and in maintaining cellular thiol-disulfide redox [5,6]. Intracellular GSH depletion is associated with upregulated levels of pro-inflammatory cytokines [7]. Cysteine (Cys) and its disulfide cystine (CySS) are the most abundant low-molecular weight thiol/disulfide redox couple in plasma [8-11], but are also present in tissues [8,11]; an oxidized Cys/CySS redox state increases pro-inflammatory cytokine production during inflammation [9,11].

Oxidative stress and cytoprotective capacity can be quantitated by determining the redox potentials of Cys/CySS and GSH/GSSG systems [8-14]. Maintained under stable, but nonequilibrium, steady-state conditions in biological systems, these redox nodes can each be disrupted, to a lesser or greater extent in plasma, tissues, and subcellular fractions depending on the type and severity of oxidative stress [5,6,8-14]. GSH has a central role as a primarily tissue antioxidant; the product of GSH oxidation, GSSG, is reduced back to GSH by GSSG reductase [5,6]. These redox pools are useful as an index of oxidative stress, as it includes components directly reflecting the availability of GSH for protection against oxidative reactions and the generation of GSSG from oxidative reactions in the correct stoichiometry [12,13]. Although Cys is regulated independently of GSH during inflammatory and other responses [9,10,15], the redox potential or reducing force (E_h_) of the GSH/GSSG couple compared to Cys/CySS E_h_ provides an integrated picture of oxidative stress within a tissue. However, to date there have been no studies exploring concomitant responses of these two redox couples in tissues in response to an oxidative challenge.

Several studies in animal models show that intestinal ischemia, radiation injury, or infection is associated with a decrease in GSH in plasma and tissues and associated organ dysfunction [3,4,16-19]. Although GSH synthesis is dependent upon the availability of cysteine, little is known regarding regulation of this precursor redox pool in tissue in response to infection. The aim of this study was to determine the concomitant regulation of tissue GSH/GSSG and Cys/CySS redox state in a model of cecal ligation and puncture (CLP)-induced peritonitis in mice.

## Methods

### Animals and experimental design

Female FVB/N strain mice (6–12 week old) were maintained on a 12 h light–dark schedule in a specific pathogen-free environment with unlimited access to standard laboratory mouse chow. All protocol procedures were approved by the Emory University Institutional Animal Care and Use Committee. A total of 16 study mice were divided into two groups (8/group): 1) mice with CLP-induced peritonitis; and 2) mice without sepsis (sham surgery as control). Mice were subjected to CLP using a 30-gauge needle, an established murine model of septic peritonitis and increased tissue proinflammatory cytokines in our laboratory [20,21]. This model results in a 27% seven-day mortality rate [20]. Briefly, anesthesia was induced and maintained with isoflurane and 5% oxygen. A small midline abdominal incision was made and the cecum exteriorized and ligated with 4–0 silk immediately distal to the ileocecal valve without causing intestinal obstruction. The cecum was then punctured once with a 30-gauge needle and a small amount of stool was extruded. The cecum was returned to the abdomen and the abdomen wall closed. Mice received 1 mL 0.9% NaCl resuscitation fluid subcutaneously immediately post-operatively and received buprenorphine, 0.1 mg/kg, post-operatively for pain control. Metronidazole (25 mg/kg, Sigma, St. Louis, MO) and ceftriaxone (0.05 mg/kg, Sigma) treatment was initiated 1 hour after CLP and repeated at 12 hours. Animals were sacrificed at 24 hours.

### HPLC analyses

Defined segments of full-thickness ileum, colon, lung and liver were derivitized with iodoacetic acid and dansyl chloride. Samples were analyzed by HPLC 24 h after laparotomy and GSH, GSSG, Cys and CySS quantitiated. The Nernst equation was used to calculate the redox potential (E_h_, in millivolts or mV) of the GSH/GSSG and Cys/CySS redox pools, respectively, in each tissue. E_h_ values that are less negative represent a more oxidized redox pool compared to more negative values.

### Quantitative real time PCR analysis

Total RNA was extracted using TRIzol (Molecular Research Center, Cincinnati, OH) from proximal ileum and colon. RNA concentration was spectrophotometrically determined at 260nm, and 1ug of total RNA was used to synthesize 10ul of cDNA using the iScript kit (Bio-Rad, Hercules, CA). Quantitative real- time PCR was performed on cDNA samples using iQ SYBR Green Supermix kit (Bio-Rad, Hercules, CA) with the Gene Amp 7000 system (PE Biosystem). Changes in relative gene expression between groups were calculated using the 2-^ΔΔCT^ method with normalization to B-actin. Specific primers were designed using the Primer Express Program (Applied Biosystems, Foster City, CA) [21]. The sequence of primers used were: 5′- AGGCTGCCCCGACTACGT- 3′ (forward) and 5′- GACTTTCTCCTGGTATGAGATAGCAAA-3′ (reverse) for TNF-α, 5′- ACAAGTCGGAGGCTTAATTACACAT-3′ (forward) and 5′- TTGCCATTGCACAACTCTTTTC-3′ (reverse) for IL-6, 5′- ACCCACACTGTGCCCATCTAC-3′ (forward) and 5′- TCGGTGAGGATCTTCATGAGGTA-3′ (reverse) for B-actin.

### Statistical analyses

Data were analyzed by unpaired *t*-test. Results (mean ± SE) were considered significant at p≤ 0.05. Pearson Regression analysis was used to associate cytokine mRNA expression and the GSH/GSSG Eh, the CyS/CySS Eh.

## Results

**Figure 1:**
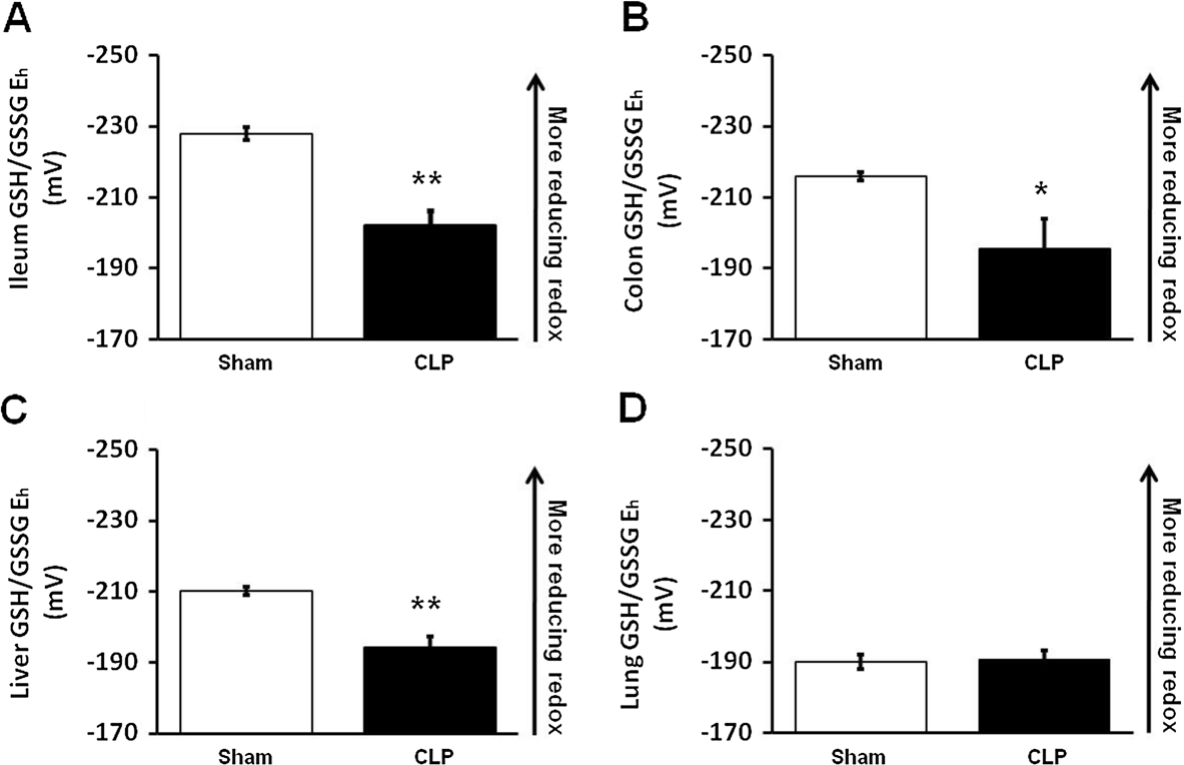
**CLP caused a more oxidized GSH/GSSG redox potential in ileum, colon and liver.** The Nernst equation was used to calculate the redox potential (E_h_, in millivolts or mV) of the GSH/GSSG redox pool in each tissue. Values are mean ± SEM, with an n=8/group. Data shown are from ileum (**A**), colon (**B**), liver (**C**), and lung (**D**) GSH/GSSG redox potential.^* =^ P< 0.05; ^**^P = < 0.01.

The CLP group exhibited a marked decrease in GSH and a modest increase in GSSG concentration in ileum (Table 1), associated with a significantly more oxidized GSH/GSSG E_h_ versus control mice (Figure 1A). In colon, CLP also induced a significant decrease in GSH and an increase in GSSG concentration (Table 1) leading to a significantly more oxidized GSH/GSSG E_h_ (Figure 1B). In liver, the CLP group showed a significant 75% decrease in GSH concentrations (Table 1). However, in contrast to the CLP response observed in ileum and colon, GSSG levels also fell markedly (50%) in response to CLP-induced peritonitis, suggesting a greater decrease in the hepatic GSH + GSSG pool size. These changes also resulted in a net oxidation of hepatic GSH/GSSG E_h_ (Figure 1C). In lung, CLP induced a modest 25% decrease in GSH concentration but a more marked decrease (50%) in GSSG concentration (Table 1). In contrast to the net oxidation of GSH/GSSG pools observed in splanchnic tissues, these changes resulted in no difference in lung GSH/GSSG E_h_ redox state between CLP-treated and control animals (Figure 1D).

**Table 1:** Tissue GSH and GSSG Concentrations

	**Ileum**		**Colon**		**Liver**		**Lung**	
	**Sham**	**CLP**	**Sham**	**CLP**	**Sham**	**CLP**	**Sham**	**CLP**
GSH	9.8±0.3	5.1±0.4^*^	6.8±0.5	4.6±0.9^*^	11.5±0.6	4.4±0.4^*^	5.8±0.7	4.2±0.3^*^
GSSG	0.2±0.0	0.3±0.1^*^	0.2±0.0	0.3±0.0^*^	0.8±0.1	0.4±0.1*	0.9±0.1	0.5±0.1^*^

Glutathione (GSH) and glutathione disulfide (GSSG) concentrations as nmol/mg protein. *CLP* cecal ligation and puncture. ^*^P< 0.05 by unpaired *t*-test.

**Figure 2:**
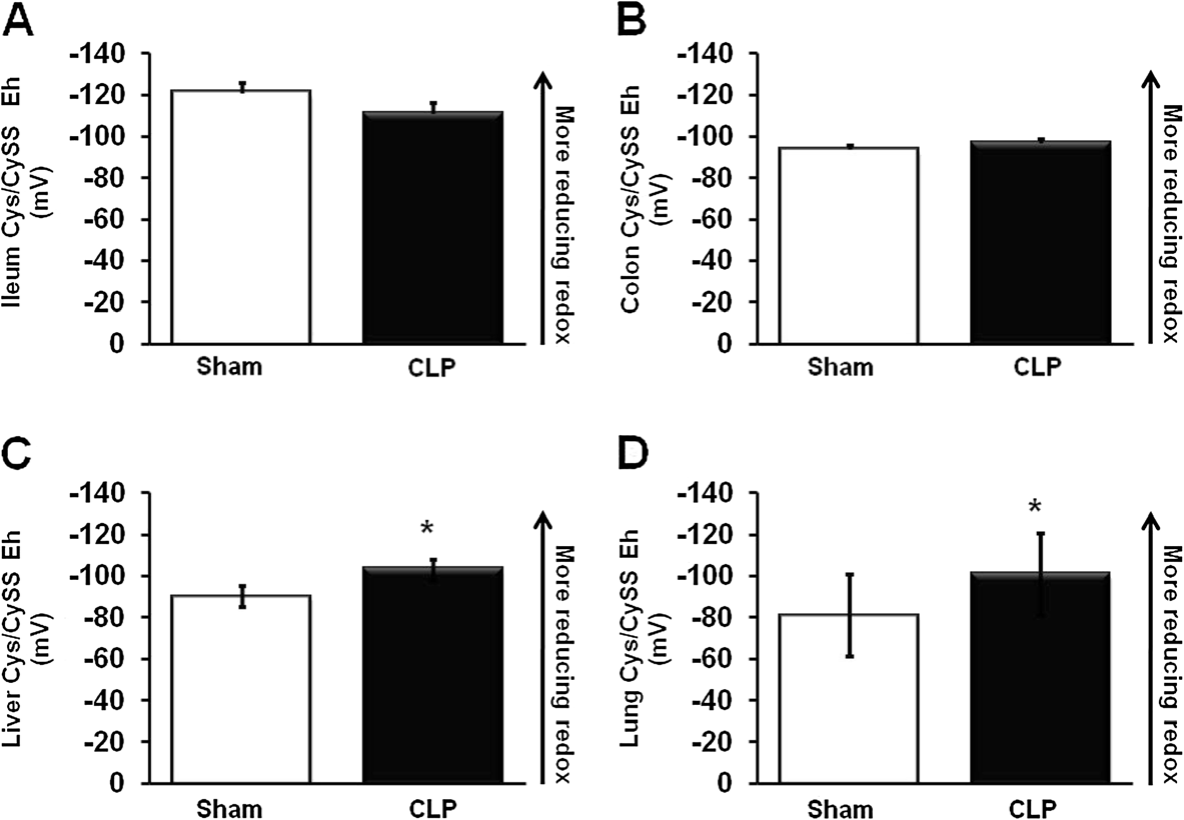
**CLP caused a more reduced E**_**h**_**Cys/CySS redox potential in liver and lung.** The Nernst equation was used to calculate the redox potential (E_h_, in millivolts or mV) of the Cys/CySS redox pool in each tissue. Values are mean ± SEM, with an n=8/group. Data shown are from ileum (**A**), colon (**B**), liver (**C**), and lung (**D**) GSH/GSSG redox potential.^* =^ P< 0.05; ^**^P = < 0.01.

Cys/CySS redox was differentially regulated in these mice compared to GSH/GSSG redox. In ileum, we observed no statistical difference in Cys or CySS concentrations or in Cys/CySS E_h_ between the CLP and control groups (Table 2 and Figure 2A). In colon, there was no statistical difference in Cys and CySS concentrations or in Cys/CySS E_h_ versus control mice (Table 2 and Figure 2B). In liver, CLP induced a 2.4-fold increase in Cys and a 1.8-fold increase in CySS concentrations, respectively (Table 2); however, in direct contrast to the marked oxidation of hepatic GSH/GSSG E_h_, the CyS/CySS E_h_ was modestly but significantly more reducing after CLP administration, probably as a result of increased Cys + CySS pool size (Figure 2C). In lung, CLP induced a 2-fold increase in Cys concentrations, while CySS remained unchanged. This resulted in a more reducing Cys/CySS redox status (Figure 2D).

**Table 2:** Tissue Cys and CySS Concentrations

	**Ileum**		**Colon**		**Liver**		**Lung**	
	**Sham**	**CLP**	**Sham**	**CLP**	**Sham**	**CLP**	**Sham**	**CLP**
Cys	0.35±0.08	0.24±0.05	0.09±0.01	0.14±0.04	0.05±0.01	0.12±0.01^*^	0.05±0.00	0.10±0.00^*^
CySS	0.17±0.01	0.20±0.02	0.13±0.02	0.19±0.07	0.06±0.01	0.11±0.01^*^	0.10±0.02	0.08±0.01

Cysteine (Cys) and cystine (CySS) concentrations as nmol/mg protein. *CLP* cecal ligation and puncture. ^*^P< 0.05 by unpaired *t*-test.

RNA expression levels of pro-inflammatory/anti-inflammatory cytokines were also measured in Ileum, colon, liver and lung. We observed no significant difference in TNF-α and IL-6 expression compared to control between tissues (Table 3). There was no significant correlation between cytokine mRNA expression and the GSH/GSSG E_h_, the CyS/CySS E_h_ (not shown).

**Table 3:** Cytokine mRNA expression in tissue

	**Ileum**		**Colon**		**Liver**		**Lung**	
	**Sham**	**CLP**	**Sham**	**CLP**	**Sham**	**CLP**	**Sham**	**CLP**
TNF-α	1.3±0.2	1.3±0.1	1.1±0.1	1.5±0.3	1.1±0.1	1.2±0.1	1.2±0.0	1.3±0.1
IL-6	ND	ND	1.2±0.0	1.4±0.2	1.5±0.4	1.3±0.2	1.3±0.2	1.2±0.0

TNF-α and IL-6 mRNA expression, normalized to β-actin, was measured via qRT-PCR in RNA extracts of Ileum, colon, liver and lung. *ND* not detectable.

## Discussion

In this study, we show that CLP-induced peritonitis differentially regulates the two major thiol-disulfide redox pools in mouse tissue. Substantial evidence has shown that sepsis and shock is associated with increased oxidative stress and depletion of tissue GSH, which in turn may contribute to organ dysfunction and impaired host response to infection [17-19]. Our results show that these redox pools are differentially regulated within and between different organs 24 hr after induction of peritonitis. CLP induced a marked 25%-50% decrease in GSH concentrations in all tissues (ileum, colon, liver and lung). In contrast, GSH disulfide (GSSG) levels increased slightly, but significantly, in ileum and colon, but decreased approximately 50% in liver and lung. These changes resulted in a significant oxidation of the GSH/GSSH E_h_ in ileum, colon and liver, but no change in redox potential of this pool in lung. Thus, oxidized GSH/GSSG E_h_ in the intestinal tissues with local peritonitis appears to be due to a combination of decreased GSH and an increase in GSSG concentrations, respectively. In contrast, the oxidation of hepatic GSH/GSSG E_h_ in this model is likely due to an overall decrease in GSH/GSSG pool size, possibly due to increased requirement for hepatic antioxidant (GSH) defense in response to bacteria and/or bacterial toxins [e.g. lipopolysaccharide (LPS), flagellin] presented to the liver via the portal circulation. Previous studies have shown that CLP induces pro-inflammatory cytokine-mediated lung injury in mice [22]. In addition, CLP-induced peritonitis in rats decreased GSH concentrations in lung in association with markers of lipid peroxidation (oxidative stress) in this tissue [23,24]. In our study, we also showed a modest, but significant decrease in GSH levels in lung in the CLP group. However, lung appeared to demonstrate an adaptive response to CLP-induced peritoneal infection/inflammation, given the concomitant decrease in GSSG resulting in no change in GSH/GSSG E_h_ (Figure 1D). In our study, we observed no change in TNF-α and IL-6 mRNA expression in lung or other tissues and no correlation with these indices and the GSH/GSSG E_h_, the CyS/CySS E_h_. Thus, local proinflammatory cytokine expression may not regulate the redox changes were observed, although possible cytokine expression changes at earlier time points after CLP may play a role.

Previous studies by our group have demonstrated that the GSH/GSSG and Cys/CySS redox pools can be differentially regulated under a variety of conditions and essentially reflect distinct redox nodes [15,25]. In HT-29 human colonic epithelial cells, we found that these thiol/disulfide redox couples were differentially regulated during extracellular oxidation [25]. In mice, we previously showed that administration of LPS oxidized Cys/CySS E_h_ in lung epithelial lining fluid, although lung tissue was not analyzed [10]. In the current study, we found that CLP-induced peritonitis did not significantly alter Cys or CySS concentrations or Cys/CySS E_h_ in ileum and colon. In contrast to responses in the two intestinal tissues, CLP resulted in a 2.4-fold upregulation in Cys level and a lesser 1.8-fold increase in CySS levels in liver, resulting in a more reducing Cys/CySS E_h_ in this tissue. In lung, CLP-induced peritonitis resulted in 2-fold increase in Cys and no change in CySS concentrations, respectively, leading to a modestly more reducing Cys/CySS E_h_ in this tissue.

The differential regulation of the thiol-disulfide redox pools in ileum, colon and liver examined in our model of infection/inflammation are consistent with in vitro studies of Jones et al. [15], who showed that under conditions of GSH depletion, the Cys/CySS redox couple is not affected, suggesting that the redox state of Cys/CySS is not directly determined by the redox state of GSH/GSSG. A limitation of our study is the lack of systemic plasma redox measures to compare with tissue changes, but in our previous studies in rats, changes in intestinal GSH/GSSG redox pools induced by altering sulfur amino acid intake in diet coincided with similar changes in this pool in plasma [8], while LPS administration in mice oxidized Cys/CySS E_h_ in lung epithelial lining fluid and in plasma [10]. To our knowledge, this is the first report on concomitant regulation of tissue GSH/GSSG and Cys/CySS redox potentials (E_h_) as markers of oxidative stress in a model of infection. In addition, we show that with CLP the lung has homeostatic mechanism(s) to prevent oxidation of the GSH/GSSG redox pool and maintains the Cys/CySS in a more reduced state. There is little information in the literature on the mechanisms that support rapid mobilization of Cys, as suggested by the increase in liver and lung after CLP in our models, but changes in the cellular transporters for CySS and/or Cys or other factors, including protein catabolism could potentially play a role [14]. Our data suggest that the GSH/GSSG redox pool in splanchnic tissue is more sensitive to oxidation in response to local peritonitis that the Cys/CySS pool in this tissue bed. Thus, translational studies on the potential cytoprotective effects of agents to upregulate the GSH pool during peritonitis (e.g. glutamine, n-acetyl-cysteine therapy, dietary sulfur amino acid supplementation) may be of interest [3,8,16]. In addition, dietary sulfur amino acid supplementation is a method to increase tissue Cys and thus improve the reducing power of the Cys/CySS redox couple in tissue and to decrease the pro-inflammatory effects of LPS mediated by interleukin-1ß [5,8,9,11,13]. Because little is known about the functional consequences of altered Cys/CySS redox state in tissue, further work is needed to understand both the regulation and role of this redox couple, including as it relates to changes in tissue GSH/GSSG E_h_, in models of infection/inflammation.

## Conclusions

Septic peritonitis induced by CLP oxidizes ileal and colonic GSH/GSSG redox but Cys/CySS E_h_ remains unchanged. In liver, CLP oxidizes the GSH/GSSG redox pool and CyS/CySS E_h_ becomes more reducing; in lung, CLP does not alter GSH/GSSG E_h_, and Cys/CySS E_h_ is less oxidized. CLP-induced infection/inflammation differentially regulates major thiol-disulfide redox pools in this murine model.

## Abbreviations

GSH: Glutathione; GSSG: Glutathione Disulfide; Cys: Cysteine; CySS: Cystine; CLP: Cecal Ligation and Puncture; HPLC: High Performance Liquid Chromatography; LPS: Lipopolysaccharide.

## Competing interests

The authors declare that they have no competing interests.

## Authors’ contributions

TRZ and CMC conceived the study, SMB, ZL, and LH collected the samples; ZL performed the CLP surgeries and maintained the mice; DPJ and YL performed redox analysis; SMB and DPJ analyzed the data; SMB and TRZ authored the manuscript; SMB, GH, TRZ, DPJ, and CMC prepared the manuscript. All authors read and approved the final manuscript.
